# The Atheist (Poetry)

**Published:** 1876-06

**Authors:** 


The fool hath said, "There is no God?"
  No God! Who lights the morning sun,
And sends him on his heavenly road,
  A far and brilliant course to run?
Who, when the radiant day is done,
  Hangs forth the moon's nocturnal lamp,
And bids the planets one by one,
  Steal o'er the night vales dark and damp?

No God! Who gives the evening dew?
  The fanning breeze, the fostering shower?
Who warms the spring morn's budding bough,
  And plants the summer's noontide flower?
Who spreads in the autumnal bower
  The fruit trees' mellow stores around,
And sends the winter's icy power
  To invigorate the exhausted ground?

No God! Who makes the bird to wing
  Its flight like arrow through the sky,
And gives the deer its power to spring
  From rock to rock triumphantly?
Who formed Behemoth, huge and high,
  That at a draught the river drains,
And great Leviathan to lie,
  Like floating isle, on ocean plain?

No God! Who warms the heart to heave
  With thousand feelings soft and sweet,
And prompts the aspiring soul to leave
  The earth we tread beneath our feet,
And soar away on pinions fleet
  Beyond the scenes of mortal strife,
With fair, etheral forms to meet,
  That tells us of the after life?

No God! Who fixed the solid ground
  Of pillars strong that alter not?
Who spread the curtained skies around?
  Who doth the oceans bounds allot?
Who all things to perfection brought
  On earth below, in heaven above?
Go ask the fool of impious thought
  Who dares to say, "There is no God!"

